# Grain-Size-Dependent Stability and Crystallographic Orientation Effects in MAFA Perovskite Thin Films

**DOI:** 10.1021/acs.jpclett.5c02728

**Published:** 2025-11-06

**Authors:** Mykhailo Khytko, Swarnendu Banerjee, Karolína Křížová, Ondřej Grundfest, Lucie Landová, Zdeňka Hájková, Karel Knížek, Robert Hlaváč, Aleš Vlk, Ema Kučerová, Antonín Fejfar, Martin Ledinský

**Affiliations:** † Institute of Physics, Academy of Sciences of the Czech Republic, Cukrovarnicka 10, 16200 Prague, Czech Republic; ‡ Faculty of Mathematics and Physics, Charles University, Ke Karlovu 3, 12116 Prague, Czech Republic; § Faculty of Nuclear Sciences and Physical Engineering, Czech Technical University in Prague, Brehova 7, 11519 Prague, Czech Republic

## Abstract

This study investigates the impact of grain size and crystallographic orientation on the stability and optoelectronic properties of MA_0.01_FA_0.99_Pb­(I_0.99_Br_0.01_)_3_ (MAFA) perovskite thin films. Grain size was tuned by varying the additive amount of methylammonium chloride (MACl) during fabrication. Macroscopic experiments demonstrated that larger grains with a dominant {100} orientation degrade rapidly under ambient conditions. In contrast, films with random orientation remain stable throughout the experiments. A maximum photoluminescence quantum yield (PLQY > 8%) was observed at an MACl molar concentration range from 0.28 mol dm^–3^ to 0.38 mol dm^–3^, which corresponded to the largest grain size with random crystallographic orientation. Beyond this range, the PLQY decreased significantly, indicating a high defect density and consequently increased nonradiative recombination, which leads to a reduced open-circuit voltage (*V*_OC,nonrad_). These findings uncover a fundamental tradeoff between grain size and long-term material stability, highlighting the critical role of crystallographic orientation control in the development of durable, high-performance perovskite solar cells.

## Graphical abstract

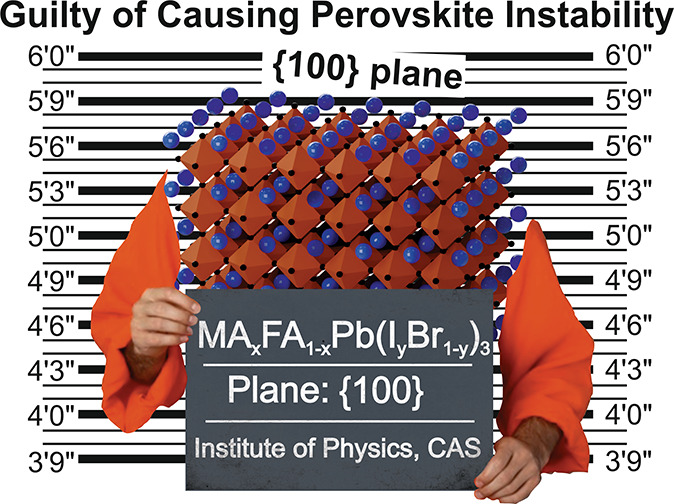

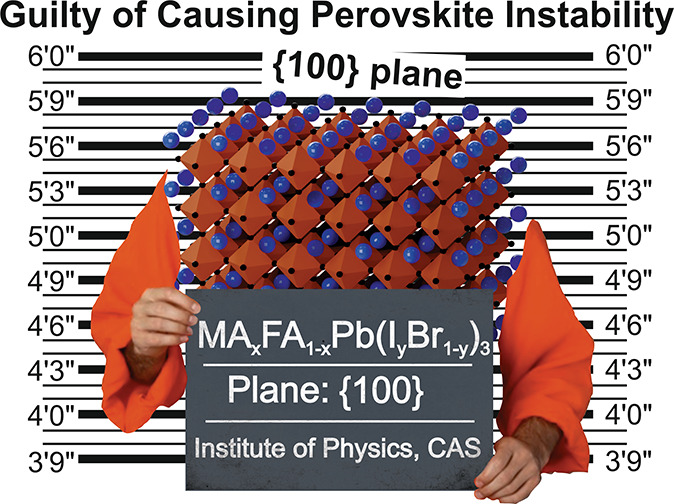

## 1 Introduction

Long-term instability is the main drawback preventing organic halide perovskite-based photovoltaics from becoming commercially viable, as they are susceptible to degradation under exposure to environmental factors such as light,^1,2^ oxygen,^3,4^ humidity,^5^ and temperature.^6^ Inspite of this, power conversion efficiency (PCE) for perovskite/Si hybrid tandem research cells reaches a remarkable 34.9%, while a single-junction formamidinium lead iodide (FAPbI_3_)-based perovskite has already reached 27%.^7^

With the increase in interest in FAPbI_3_ films to achieve high PCE, many studies continue to use methylammonium chloride (MACl) as an additive to enhance the phase stability of the photoactive α-FAPbI_3_ phase.^8–13^ The inclusion of MACl promotes the formation of larger grains and reduces defect density within the film.^14^ The residual MA^+^ leads to the formation of mixed-cation (MAFA) compositions, deviating from the pure FAPbI_3_ phase.^8,14^ MAFA films exhibit a polycrystalline morphology with individual grains separated by distinct grain boundaries. Such a film facilitates rapid and efficient transfer of photogenerated charge carriers for the final device.^15,16^

Although larger grains are generally favored for suppressing nonradiative recombination and enhancing charge transport, the relationship between grain size and stability is more complex than previously assumed. Some studies suggest that smaller grains degrade more quickly due to their higher grain-boundary density, which facilitates rapid ion migration and accelerates degradation under environmental stressors.^17^ Conversely, larger grains can also be prone to rapid degradation: films with grain sizes exceeding 2 μm have shown improved efficiencies but still exhibited stability challenges, indicating that grain size alone is insufficient to guarantee durability.^18^ Moreover, crystallographic orientation appears to play a decisive role—films with misaligned or unfavorable faceting may exhibit surface chemistries and ion migration pathways that compromise stability.^19,20^

This study investigates the grain-size-dependent degradation of MAFA perovskite, highlighting the correlation between grain size, crystallographic orientation, film stability, and optoelectronic quality. Grain size and the density of grain boundaries were characterized using atomic force microscopy (AFM) and scanning electron microscopy (SEM). Crystallographic orientation was determined via X-ray diffraction (XRD) analysis. Film stability was qualitatively assessed through observable color changes. Variable grain sizes were achieved by modulating the concentration of MACl in the precursor solution during the perovskite film fabrication process. Photoluminescence quantum yield (PLQY) measurements identified an optimal MACl concentration that produced the highest PLQY. A high PLQY value corresponds to higher radiative recombination in the film, leading to reduced open-circuit voltage (*V*_oc_) losses, which is desirable for improved device performance.

## 2 Methods

### 2.1 Preparation of the Films

To prepare MAPbBr_3_ crystals, 4.4041 g of lead bromide (PbBr_2_, Sigma–Aldrich) and 1.3436 g of methylammonium bromide (MABr, Greatcell Solar) were codissolved in 10 mL of dimethylformamide (DMF, anhydrous, Sigma–Aldrich) at room temperature. Subsequently, the solution was filtered using a PTFE filter with 0.45 μm pore size. Perovskite crystals started to grow by heating the filtered solution overnight at 100–130 °C. To prepare the perovskite films, the precursor solution was prepared by dissolving 0.9679 g of lead iodide (PbI_2_, >98.0%, TCI), 0.3611 g of formamidinium iodide (FAI, Greatcell Solar), and 0.0080 g of MAPbBr_3_ crystals in 0.8 mL DMF (anhydrous, Sigma–Aldrich) and 0.2 mL dimethyl sulfoxide (DMSO, anhydrous, Sigma–Aldrich). Nine FAPbI_3_ precursor solutions with varying amounts of methylammonium chloride (MACl, Greatcell Solar) were prepared. The MACl molar concentrations, approximated based on estimated solution volumes, range from 0 to 1.04 mol dm^–3^ (see ). The solutions were filtered using a 0.2 μm PTFE filter. Glass substrates were sequentially cleaned in acetone (Penta) and isopropanol (IPA, Penta) for 15 min each in an ultrasonic bath, followed by oxygen plasma treatment (100 W, 120 s). For each sample, 70 μL of the filtered solution was spread over the glass substrate and spin-coated at 6000 rpm for 50 s. During spin coating, 1 mL of diethyl ether (Lachner) was dripped onto the film after spinning for 5–10 s. Finally, the resulting perovskite film was annealed on a hot plate at 150 °C for 15 min, followed by another annealing at 100 °C for 30 min. Following crystallization, the incorporated MA content was assumed to be less than 10% of the initial value, consistent with the findings reported by Zheng et al.^21^

### 2.2 Characterization

#### 2.2.1 Atomic Force Microscopy (AFM)

AFM measurements were conducted using a Bruker Dimension ICON system operating in PeakForce Quantitative Nanomechanical mode. A Multi75Al-G probe (Budget Sensors) with an aluminum coating on the reflective side of the cantilever was employed. The tip has a nominal radius of 10 nm and exhibits half-cone angles of 20°–25° along the cantilever axis, 25°–30° from the side, and 10° at the apex. Images were acquired at a scan rate of 0.1 Hz with 512 points per line. Image processing was performed with Gwyddion, and the lateral grain dimensions were subsequently determined using ImageJ.

#### 2.2.2 Scanning Electron Microscopy (SEM)

The surface morphology of the perovskite films was characterized using ultrahigh-resolution SEM. All samples were examined with a Tescan MAIA3 SEM, equipped with a Schottky emitter and operating in a high vacuum. Images were acquired using a secondary electron detector at an accelerating voltage of 5–10 kV. The lateral grain sizes were quantified using ImageJ software.

#### 2.2.3 Photoluminescence Quantum Yield (PLQY)

A custom in-house-designed PLQY measurement setup was employed to determine the photoluminescence quantum yield. The setup consists of an integrating sphere (Thorlabs, general-purpose 50-mm-diameter integrating sphere), a light source (LED, Thorlabs, Model M455L4, 455 nm, with an intensity of approximately one sun), two silicon photodiodes for the simultaneous detection of emitted and absorbed light, and a long-pass filter (Thorlabs edgepass filter, Model FELH0500).

#### 2.2.4 Photoluminescence (PL)

The photoluminescence (PL) spectra were acquired using a Renishaw InVia confocal spectrometer equipped with 532-nm laser excitation and a Leica 50× objective inside a nitrogen glovebox, providing PL mapping over 40 μm × 40 μm areas. To ensure the samples remained pristine, they were hermetically transferred between gloveboxes, sealed in aluminum foil within an airtight jar under a nitrogen atmosphere, thereby avoiding exposure to light and ambient air. During the PL measurements, the samples showed no signs of degradation under 532-nm excitation.

#### 2.2.5 X-ray Diffraction (XRD)

XRD using a Bruker D8 Advance diffractometer (CuK radiation, Lynxeye XE-T position-sensitive detector) was employed to determine the phase compositions, preferred orientation, and crystallite sizes.

## 3 Results and Discussion

We investigated a set of MA_0.01_FA_0.99_Pb­(I_0.99_Br_0.01_)_3_ (MAFA) perovskite samples with additive amounts of MACl to optimize their composition for further optoelectronic applications. MACl concentration in the precursor solutions was varied in the range of 0–1.04 mol dm^–3^. AFM and SEM imaging (Figure 1, ) demonstrate the progression of lateral grain size and grain-boundary length with increasing MA^+^ content in MAFA samples. This trend is further highlighted in Figure 1j, which clearly illustrates the consistent growth in average grain size measured by both techniques. Incorporation of MA^+^ into the precursor alters the crystallization dynamics of FAPbI_3_, promoting the conversion from the nonperovskite δ-FAPbI_3_ phase to the conductive α-FAPbI_3_ phase, resulting in varied grain sizes.^22,23^ Changes in MA^+^ concentration not only affect grain size but also decrease grain boundary length, which contains defects responsible for nonradiative recombination of charge carriers in the perovskite layers.^24–28^

**Figure 1:**
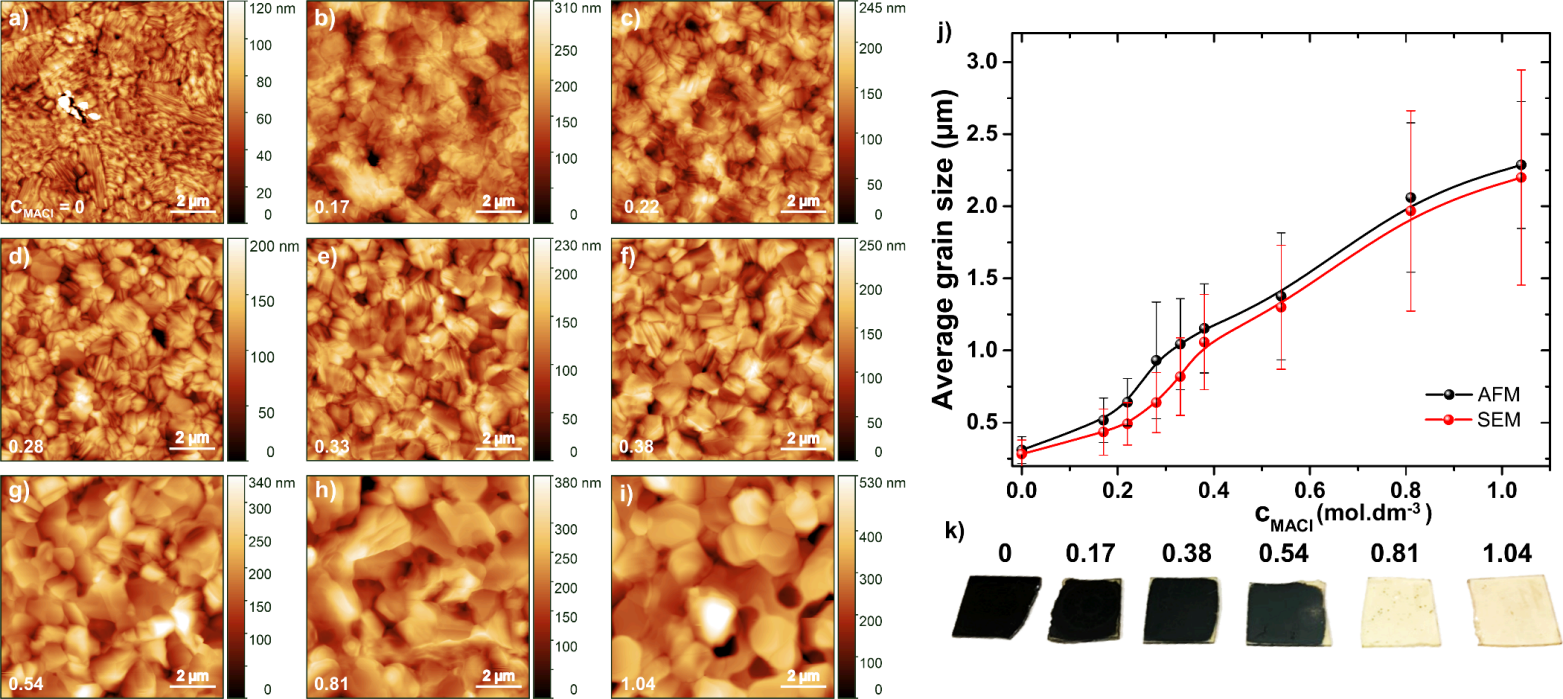
AFM images of MAFA thin films, synthesized with varying MA^+^ molar concentrations, reveal corresponding modifications in grain size distribution, where *c*_MACl_ [mol dm^–3^] = (a) 0, (b) 0.17, (c) 0.22, (d) 0.28, (e) 0.33, (f) 0.38, (g) 0.54, (h) 0.81, and (i) 1.04. (j) Average grain size distribution of the films as a function of the MACl content. (k) Optical photograph of samples with different MACl concentrations after 72 h of exposure to ambient conditions.

In order to address the degradation issue, we investigated the environmental stability of the films under ambient conditions. The picture taken after 72 h of exposure (Figure 1k) shows that with increasing MACl concentration, the film is significantly more prone to degradation, as indicated by rapid discoloration to yellow. This contradicts previous findings by Wang et al., who reported a linear correlation between degradation time and grain size in MAPbI_3_, with smaller grains degrading faster due to the increased density of grain boundaries.^29^ Our observation suggests a more complex interplay between grain size, environmental stability, and other factors.

Larger grain sizes are generally favored in photovoltaic applications to minimize defects in grain boundaries, thereby maximizing *V*_OC_ and enhancing charge carrier transport.^30^ However, our findings indicate that grains exceeding ∼1.3 μm average lateral dimensions (Figures 1j and 1k) are highly susceptible to degradation, despite their lower grain-boundary density. This observation suggests that additional factors play a critical role in determining long-term structural stability.

One of the factors may be crystallographic orientation. Therefore, we performed XRD analysis on the as-deposited MAFA thin films (Figure 2a), covering a 2θ range from 10° to 70°. The XRD pattern of the pristine samples shows reflections that match specific crystallographic planes of the cubic mixed-cation perovskite α-phase.^31^ This confirms the formation of the photoactive perovskite phase and the absence of nonperovskite phases in the as-prepared films. In contrast, the XRD pattern of the sample (*c*_MACl_ = 0.81) degraded in air after 72 h of exposure shows a transition from the pure α-phase to approximately 85 vol % δ-phase and 15 vol % residual α-phase. Additionally, it clearly reveals the evolution of preferential grain orientation with varying *c*_MACl_ variation (Figure 2b).

**Figure 2:**
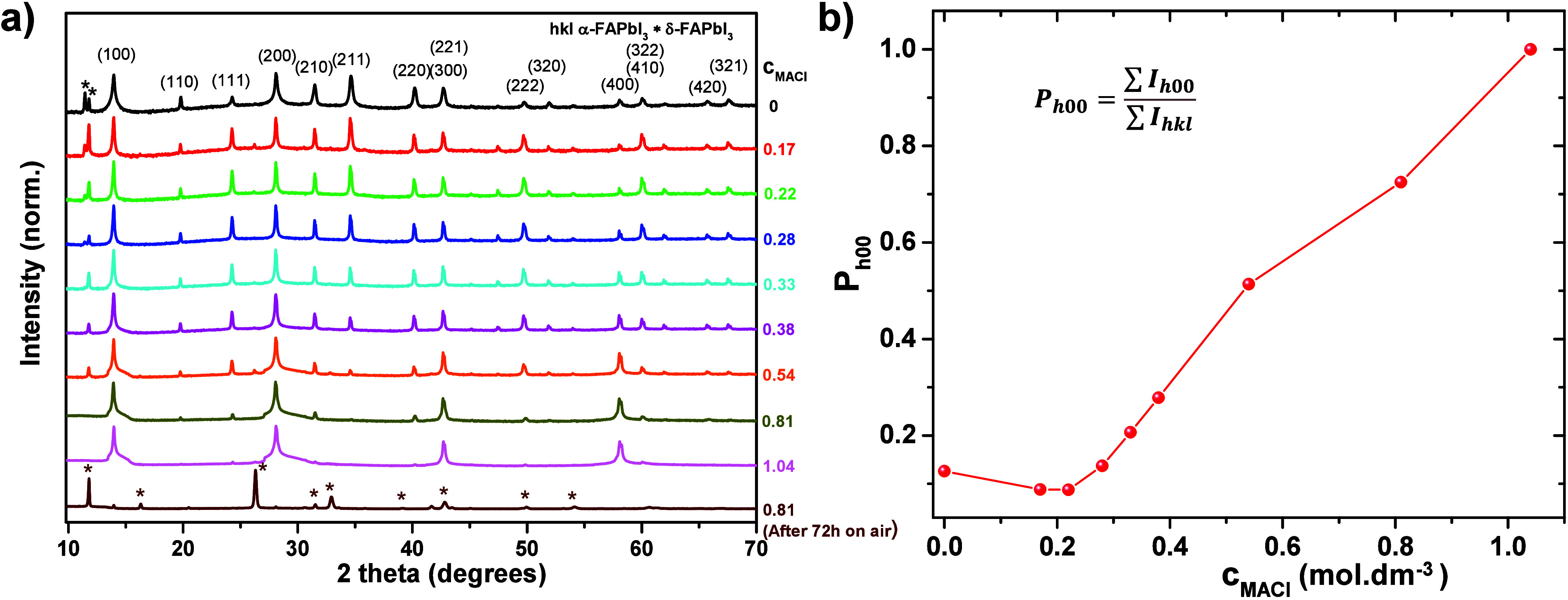
(a) X-ray diffraction (XRD) patterns of MAFA thin films as a function of varying molar concentration of MACl in a linear intensity scale. Key reflections corresponding to α-FAPbI_3_ and δ-FAPbI_3_ impurity phases are indexed. (b) Analysis of XRD data reveals a correlation between the integrated intensity of {100} crystallographic planes and the MACl concentration during the fabrication of FAPbI_3_ thin films, indicative of preferential orientation evolution.

In Figure 2a, we observe a variety of peaks, including {100}, {110}, and {111}. Each of these notations represents a group of reflections—for example, {100} includes (100), (200), (300), and (400), while {110} and {111} similarly represent their corresponding families of planes. The shoulders of the {100} and {200} peaks in Figure 2 are exaggerated for higher MA+ concentrations due to the logarithmic intensity scale. On a linear scale, the peaks display a standard shape that can be well-described by pseudo-Voigt or Lorentzian profiles, and they are essentially symmetric. With increasing MA^+^ concentration, the intensities of all grain orientations begin to decrease, except for {100}, which becomes dominant. We define the degree of {100} crystallographic orientation (*P*_*h*00_), as shown in Figure 2b, as the ratio of the sum of the reflection intensities along the {100} directions to the sum of intensities from all crystallographic orientations, according to the following equation:

$$P_{h00}=\sum I_{h00}/\sum I_{\mathrm{hkl}}$$ 1

Figure 2b shows that the initial increase in MA^+^ molar concentration up to *c*_MACl_= 0.28 mol dm^–3^ does not reveal preferential orientation in the {100} crystallographic direction. However, a further increase in MA^+^ molar concentration enhances the contribution corresponding to the {100} crystallographic plane (Figure 2b). This suggests a correlation in which a higher degree of {100} crystallographic orientation is associated with larger grains that undergo a faster degradation (Figure 1k), indicating reduced stability.

PLQY is a key parameter for evaluating the efficiency of radiative recombination in semiconductor materials, providing insight into the balance between radiative and nonradiative processes. Figure 3 illustrates the PLQY of the MAFA thin films as a function of *c*_MACl_. We observe an increase in the PLQY signal up to *c*_MACl_ = 0.33 mol dm^–3^, followed by a decrease. If we increase the grain size (*c*_MACl_ from 0 to 0.33 mol dm^–3^), we observe an increase in the PLQY due to a reduction in the number of defects at grain boundaries that cause nonradiative recombination. Surprisingly, a further increase in grain size (*c*_MACl_ from 0.33 to 1.04 mol dm^–3^) results in a decrease in PLQY, despite the continued reduction in grain-boundary length and, consequently, defect density. This behavior is not related to grain boundaries but rather arises from the increased presence of grains with {100} crystallographic orientations (Figure 2b).

**Figure 3:**
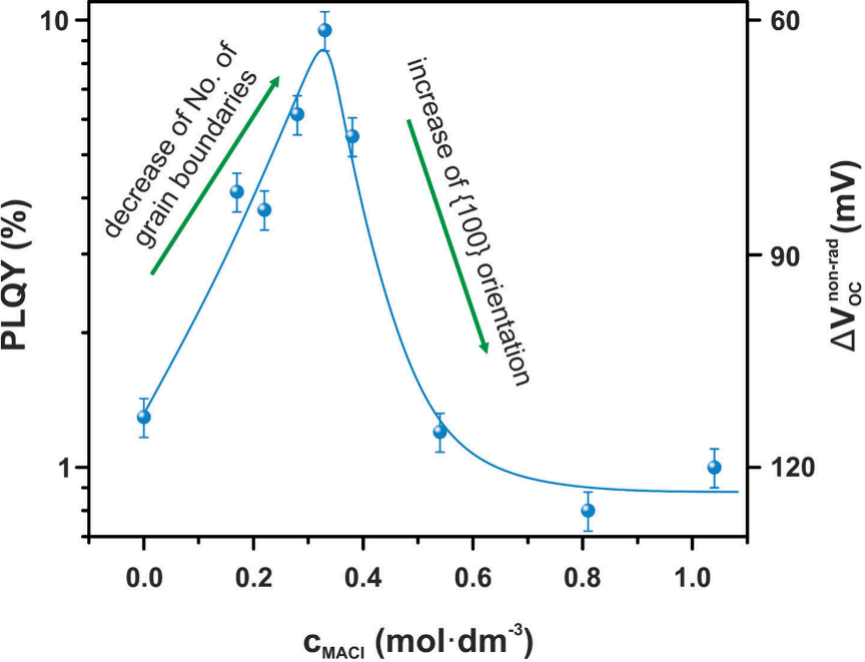
PLQY as a function of the molar concentration of MACl in MAFA thin films, illustrating the correlation between MACl content and radiative recombination efficiency. The blue line is present as a guide for the eyes.

PLQY is quantitatively related to the nonradiative voltage loss (Δ*V*_OC,nonrad_) through the expression Δ*V*_OC,nonrad_ = −*kT* ln­(PLQY).^32^ In device engineering, minimizing Δ*V*_OC,nonrad_ by improving the PLQY is crucial for enhancing solar cell efficiency, as nonradiative pathways directly reduce the output power by lowering the voltage.^32,33^ The PLQY signal, shown on the graph in Figure 3, indicates that optimizing the concentration of MA^+^ can yield a *V*_OC_ gain of 60 mV.

Additionally, PL mapping over 40 μm × 40 μm areas reveals a small blue shift of approximately 15 meV in the PL peak when the MACl concentration is increased from 0 to 0.3 mol/dm^3^ (). At higher concentrations, however, both the PL peak position and the bandgap remain unchanged, and the FWHM of the PL spectra is constant within experimental error. This invariance in PL response indicates that neither MA^+^ nor Cl^–^ are significantly incorporated into the perovskite lattice beyond 0.3 mol/dm^3^. Consequently, the perovskite films across the series are compositionally similar, and the observed differences in stability can be attributed primarily to changes in crystallographic orientation rather than to variations in material composition.

While larger grains typically reduce grain-boundary density and thereby enhance optoelectronic performance, as evidenced by higher PLQY values, our results indicate that films with dominant {100} facets exhibit accelerated environmental degradation. This facet-dependent behavior arises from two complementary effects: (i) {100} surfaces possess higher surface defect densities, which can increase nonradiative recombination under illumination; and (ii) these surfaces are more chemically “active,” rendering them more susceptible to external factors such as moisture and oxygen.^34–37^ Consequently, PLQY measurements primarily reflect the intrinsic potential for high photovoltaic performance, whereas ambient degradation highlights the facet-dependent susceptibility of the material. This distinction underscores the importance of considering both grain size and crystallographic orientation when optimizing MAFA perovskite thin films for stable, high-efficiency devices.

## 4 Conclusions

This study identifies crystallographic orientation as a critical determinant of both stability and optoelectronic performance in MAFA perovskite films. We demonstrate that the {100} orientation accelerates degradation under ambient conditions, whereas its suppression leads to significantly enhanced environmental stability. Degradation of samples with preferential {100} orientation under ambient conditions confirms that this orientation has a higher defect density, as further supported by the reduced PLQY signal. Grain size, modulated by MACl concentration, was found to influence crystallographic orientation, revealing a clear relationship among grain size, orientation, and environmental stability. Notably, films with an intermediate MA^+^ molar concentration (*c*_MACl_ from 0.28 mol dm^–3^ to 0.38 mol dm^–3^) exhibited large grains with random crystallographic orientation and achieved peak PLQY values (>8%), indicating a reduced defect density and suppressed nonradiative losses. These results highlight a fundamental tradeoff: while grain size improves certain optoelectronic properties, it must be balanced against the risk of stability loss from unfavorable orientation. Together, these insights offer a materials design strategy for tuning crystallography and composition to achieve stable, high-efficiency perovskite photovoltaics.

## Supplementary Material





## Notes

Supplemental figures and tabular data (PDF)
Transparent Peer Review report available (PDF)
