# Supplementary material for: Grain-Size-Dependent Stability and Crystallographic Orientation Effects in MAFA Perovskite Thin Films
Source: J Phys Chem Lett. 2025 Nov 6;16(46):11901–6. doi: 10.1021/acs.jpclett.5c02728 (PMC12641472; doi:10.1021/acs.jpclett.5c02728)
Supplement: Supplementary file 1 [file jz5c02728_si_001.pdf]

Supporting Information:
Grain Size-Dependent Stability and Crystallographic Orientation Effects in MAFA Perovskite Thin Films

Mykhailo Khytko[1,2], Swarnendu Banerjee[1,3], Karolína Křížová[1], Ondřej Grundfest[1], Lucie Landová[1], Zdeňka Hájková[1], Karel Knížek[1], Robert Hlaváč[1,3], Aleš Vlk[1], Ema Kučerová[1], Antonín Fejfar[1], Martin Ledinský[1].

1 - Institute of Physics, Academy of Sciences of the Czech Republic, Cukrovarnicka 10, 16200 Prague, Czech Republic.

2 - Faculty of Mathematics and Physics, Charles University, Ke Karlovu 3, 12116 Prague, Czech Republic.

3 - Faculty of Nuclear Sciences and Physical Engineering, Czech Technical University in Prague, Brehova 7, 11519 Prague, Czech Republic.

1. The figure below presents the gradual evolution of grain sizes in $MA_{0.01}FA_{0.99}Pb(I_{0.99}Br_{0.01})_3$ (MAFA) perovskite samples with varying MA^+ concentrations, as observed from top-view SEM images.

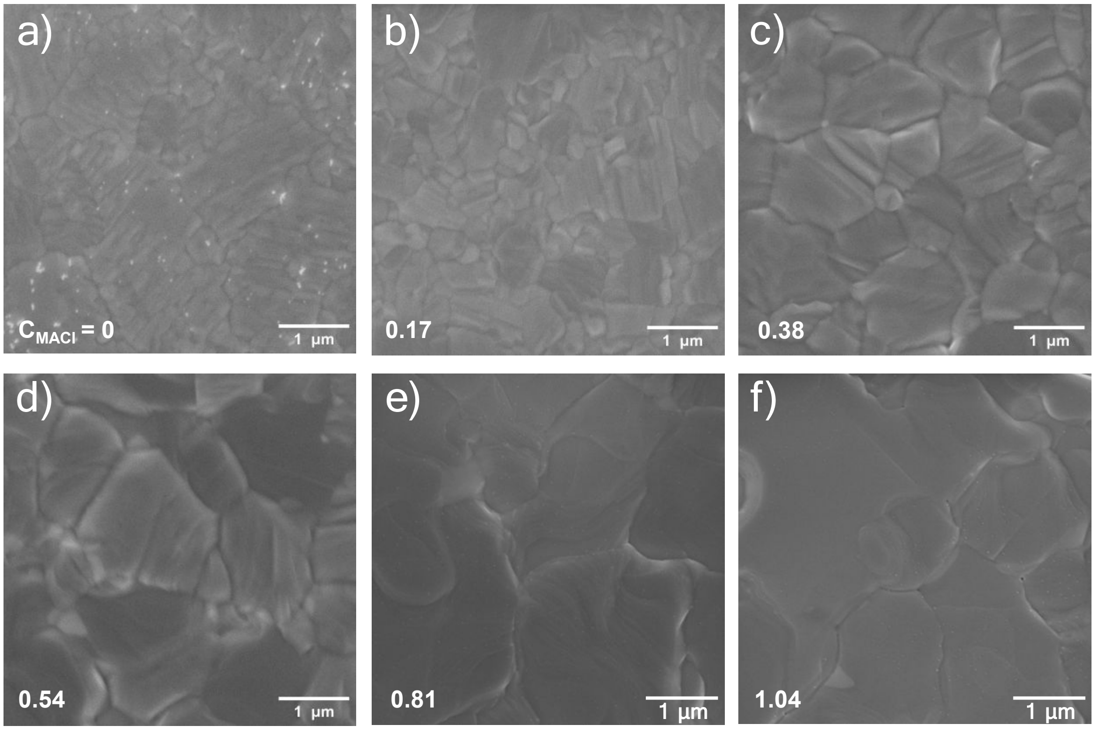

Fig. S1: Evolution of grain sizes in MAFA perovskite films. SEM images (top view) of MAFA films achieved by modulating the molar concentration of MACl [$mol \cdot dm^{-3}$] in the precursor solutions: a) pristine (without addition), b) 0.17 c) 0.38 , d) 0.54 , e) 0.81 , and f) 1.0

2. The figure below shows how the PL peak position and FWHM vary with the concentration of MA^+ in the initial precursor solution. As the PL response remains nearly unchanged beyond a certain MA^+ concentration, it suggests that MA incorporation does not induce significant structural changes. Therefore, our assumption that crystallographic orientation is the primary driving force behind observed degradation is well justified.

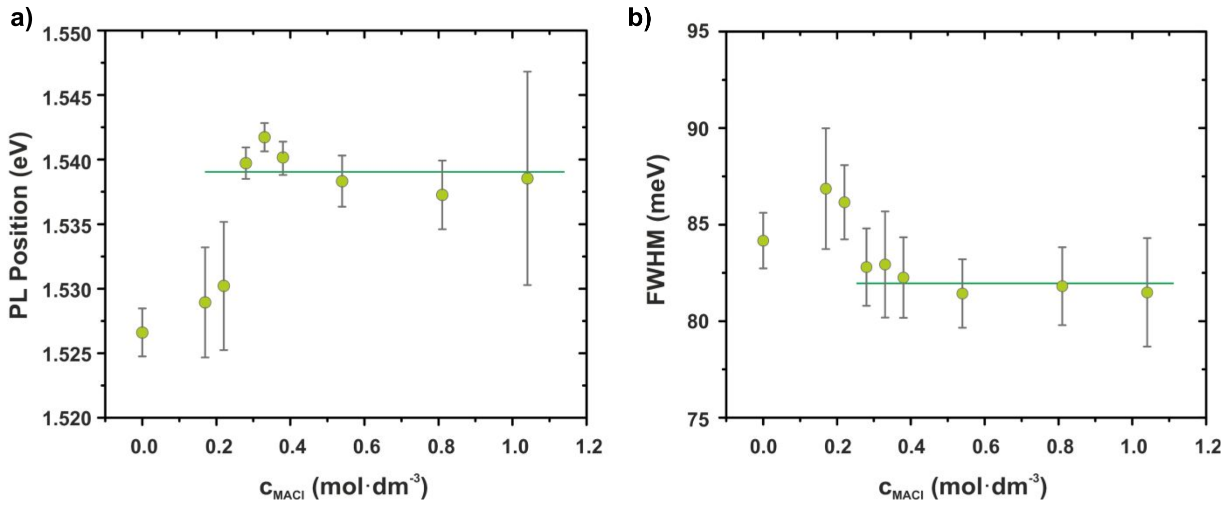

Fig. S2: PL peak position (a) and full width at half maximum (b) as a function of the molar concentration of MACl in MAFA thin films. The green line is the guide for the eyes.

Sample Preparation and Molar Concentration Approximation

Nine solutions were prepared by adding varying amounts of methylammonium chloride (MACl) to 430 μl aliquots of the initial mixed precursor solution. The masses of MACl added ranged from 0 mg to 32.9 mg per sample.

- The volume of MACl added was calculated assuming the crystalline form's density as 1.1 g/cm³.

- The final volume was considered as the sum of the original solution volume and the volume of added crystalline MACl.

Minor volume contraction or expansion effects were not taken into account, so the concentrations are approximate.

Sample №	MACl added [mg]	Considered Volume[µl]	MACl molar concentration [mol·dm^{-3}]
1	0	430	0
2	4.9	434	0.17
3	6.6	436	0.22
4	8.2	437	0.28
5	9.9	439	0.33
6	11.5	440	0.38
7	16.4	445	0.54
8	24.7	452	0.81
9	32.9	460	1.04

Table S1: MACl was added to $FAPbI_3$ precursor solutions at different masses as listed. Approximate final solution volumes were calculated from solid densities, neglecting volume changes upon mixing. The reported molar concentrations are therefore approximate.
