# Supplementary material for: Grain-Size-Dependent Stability and Crystallographic Orientation Effects in MAFA Perovskite Thin Films
Source: J Phys Chem Lett. 2025 Nov 6;16(46):11901–6. doi: 10.1021/acs.jpclett.5c02728 (PMC12641472; doi:10.1021/acs.jpclett.5c02728)
Supplement: Supplementary file 2 [file jz5c02728_si_002.pdf]

jz-2025-027282.R1

Name: Peer Review Information for "Grain Size-Dependent Stability and Crystallographic Orientation Effects in MAFA Perovskite Thin Films"

First Round of Reviewer Comments

Reviewer: 1

Comments to the Author

Comments on manuscript#jz-2025-027282

" Grain Size-Dependent Stability and Crystallographic Orientation Effects in MAFA Perovskite Thin Film"
By Mykhailo Khytko et al.

This manuscript describes a study on the influence of grain orientation in terms of photocarrier recombination and stability of double cation perovskite materials, combining XRD and PL measurements.

The manuscript concludes in particular that, for this type of mixed methylammoniumformamidinium perovskite, the predominance of (100) facets is detrimental in terms of performance and leads to a material that is more sensitive to the environment, and therefore whose electronic properties degrade more quickly.

This conclusion is maybe not really new, as evidenced by several studies cited in the references in the manuscript. However, the work presented here provides new experimental evidence through the use of XRD and photoluminescence techniques. Furthermore, the manuscript is well and clearly written, and my opinion is that it deserves publication. However, there are a few points that are not fully clear and/or should be developed. These should be addressed before the manuscript can be published.

1) The samples are stated as $MA_{0,01}FA_{0,99}Pb(I_{0,99}Br_{0,01})_3$. However, since additive amounts of MACl are used in the precursor solutions, one can wonder if the composition of the perovskite has changed or not. This should be precised.

2) Related to the preceding remark, is there any change in the photoluminescence spectrum when changing the MACl content in the solution? Photoluminescence measurements are summarized in terms of PLQY, but there it would be nice to have the corresponding PL spectra (photon energy or wavelength dependence) to appreciate possible changes in bandgap energy or presence/appearance of diUerent phases.

3) Clarification is also needed regarding the conditions and protocols for photoluminescence measurements. In particular, as the probed materials are highly sensitive to air and can degrade quite quickly, it is important to carry out measurements as soon as possible after deposition, and before any degradation occurs. Can you give some precisions? (and also precise if the samples do not degrade upon luminescence measurements)

4) The description of X-ray diUraction patterns could be somewhat developed. Indeed, you focus on the predominance of {100} related peaks and disappearance of other peaks when increasing C_{MACl} beyond 0.38. However, one can also observe that at the same time the peaks related to the {100} family become strongly asymmetric with a pronounced shoulder on the high 2q side of the peak. I think that it is important to comment on that and propose some explanation because this might also be an indicator of the increased sensitivity to degradation of the samples under ambient conditions, not only just the predominance of {100} facets.

5) Also, there are peaks at low 2q values (<15 degrees) marked with stars. What is the origin of these peaks? What is the meaning of the marked stars? You should add information on that (at least in the figure caption)

6) Do you have some information on possible strain in the material and its relation to stability issues?

7) Stability is only considered from visual pictures of the samples after 72 hours. There is no indication about structural and photoelectrical changes of the material. Is the evolution linked to the appearance of the d phase or to PbI_2? The d phase should be visible in the Xray diUraction patterns. Did you investigate the samples through XRD and PL after the 72 hours storage period?

8) One of the main concern is the distinction between performance and stability. Indeed, PL measurements are an indicator of quasi-Fermi level splitting, which can be linked to the open-circuit voltage in cells. This is related to the potential performance of solar cells. So the direct comparison of PL and XRD establishes a link between the structural properties, in particular the presence of (100) facets, and performance. On the other hand, you observe that samples prepared with larger MaCl content in the solution exhibit stronger visual degradation. But the conclusion drawn here is not straightforward and mixes performance and stability. Larger grains are normally supposed to be favourable to performance due to less grain boundaries and therefore less recombination activity. The presented results do not agree with this general thought, since the grain size continuously increases with the MaCl content. It is maybe possible to discuss the measurement results and this usual guess in terms of surface states. One could also relate the higher sensitivity of (100) surfaces to surface activity. In other words, could your combined measurements point toward both a larger surface recombination detrimental to performance (due to higher surface defect density or to higher capture cross sections of the surface defects) and a larger surface "activity" or sensitivity to external factors detrimental to stability

9) In the conclusion, the range of c_{MACl} allowing to achieve PLQY larger than 8% is stated as from 0.54 and 0.81 mol dm^{-3}. This is not in agreement with the results of Fig. 3 and with the abstract and should be corrected to "from 0.28 to 0.38 mol dm^{-3}".

Reviewer: 2

Comments to the Author

In this study from Khytko and coworkers the impact of grain size and crystallographic orientation on the stability and optoelectronic properties of FAMA perovskite thin films was investigated.

By varying the amount of MACl in the spin-coating solvent the grain sizes could be gradually increased. Color changes demonstrate that larger grains with a dominant {100} orientation degrade more rapidly under ambient conditions than isotropic aligned samples with small grains.

At present studying the stability and revealing the degradation mechanism are very important topics for the use of perovskite materials. As such the present manuscript is of interest for JPCL. At the same time the main message of the paper is for me not very well argued. For this reason the manuscript is at this stage not appropriate for JPCL. The hesitations I have are as follows:

- All effects are related to the preferential orientation of the PVK, however in current literature the presence of MA negatively affects the stability. The composition of the PVKs with different amounts of MACl also changes which could also explain the degradation. Also Cl- could negatively affect the stability.

- With no or little amount of MACl the gamma phase is observed in the XRD patterns. It could also be argued that a small amount of gamma phase suppresses degradation.

- For PVK's with a large amount of MACl the XRD peaks for the (100) and (200) show a very broad base. What causes this? Could that be the factor of importance for the stability?

other issues:

- What are the resulting compositions for the different additions in the PVK film? (ratio MA to FA? Cl?)

- The degradation assessment is very basic. Just having a couple of absorption spectra measured at different days could already provide a lot of detailed information.

- Recording the XRD during degradation could also confirm the claims of the manuscript

- Muscarella et al have a relevant paper on this topic. (J. Phys. Chem. Lett. 2019, 10, 6010−6018)

Author's Response to Peer Review Comments:

Dear Editor and Reviewers,

We would like to sincerely thank both reviewers for their time, effort, and valuable comments, which have helped us to substantially improve and clarify our manuscript.

Please find attached the following documents for your consideration:

Revised_Manuscript_ACSLetters

Revised_Manuscript_With_Changes_ACSLetters

Response_to_Reviewers_ACSLetters

All files are provided in both docx and pdf formats.

We hope that the revisions and clarifications address all reviewer comments satisfactorily.

Thank you for your consideration.

Best regards,

Mykhailo Khytko

Reviewer #1

Original review: *This manuscript describes a study on the influence of grain orientation in terms of photocarrier recombination and stability of double cation perovskite materials, combining XRD and PL Measurements.*

The manuscript concludes in particular that, for this type of mixed methylammonium-formamidinium perovskite, the predominance of (100) facets is detrimental in terms of performance and leads to a material that is more sensitive to the environment, and therefore whose electronic properties degrade more quickly.

This conclusion is maybe not really new, as evidenced by several studies cited in the references in the manuscript. However, the work presented here provides new experimental evidence through the use of XRD and photoluminescence techniques. Furthermore, the manuscript is well and clearly written, and my opinion is that it deserves publication. However, there are a few points that are not fully clear and/or should be developed. These should be addressed before the manuscript can be published.

Dear Reviewer 1, thank you for your time and the valuable comments, questions, and suggestions. The answers are listed below.

1) **The samples are stated as $MA_{0,01}FA_{0,99}Pb(I_{0,99}Br_{0,01})_3$. However, since additive amounts of MACl are used in the precursor solutions, one can wonder if the composition of the perovskite has changed or not. This should be precised.**

We thank the reviewer for raising this important point regarding the final composition of the perovskite films. Indeed, determining the precise stoichiometry after film formation remains a well-known challenge in this field. We attempted to characterize the composition using NMR; however, the results could not be interpreted reliably in this case.

Despite the large excess of MACl in the precursor solution, the amount of MA^+ incorporated into the final perovskite lattice is strongly reduced during crystallization and particularly during thermal annealing, due to the volatile nature of MA^+. Zheng et al. (ref. 1) demonstrated that even when using precursor solutions with significantly higher MACl contents, the residual MA^+ fraction stabilizes at only ~2–3 mol% of the A-site cations after optimized annealing. Consistent with this, we assumed that in our films the incorporated MA^+ content remains below 10% of the initial precursor amount. This strongly supports our conclusion that the final layers contain only a very minor fraction of MA^+, and that the observed effects are dominated by structural orientation rather than by a major change in bulk composition.

For clarity, we report both the initial precursor solution composition and the use of MACl additives (Table S1), as well as the detailed sample preparation procedure described in Section
2.1 of the main manuscript.

Ref. 1 - Zheng, D.; Chen, F.; Rager, M.; Gollino, L.; Zhang, B.; Pauporté, T. What Are Methylammonium and Solvent Fates upon Halide Perovskite Thin-Film Preparation and Thermal Aging? *Adv. Mater. Interfaces* **2022**, *9* (32). https://doi.org/10.1002/admi.202201436.

2) **Related to the preceding remark, is there any change in the photoluminescence spectrum when changing the MACl content in the solution? Photoluminescence measurements are summarized in terms of PLQY , but there it would be nice to have the corresponding PL spectra (photon energy or wavelength dependence) to appreciate possible changes in bandgap energy or presence/appearance of different phases.**

Thank you for this valuable comment. During PLQY measurements, we used a Keithley oscilloscope, which outputs the integrated area under the PL curve; therefore, individual PL spectra were not collected in that setup. However, we performed PL mapping on all

samples over 40 × 40 μm^2 areas using a Renishaw InVia confocal spectrometer operating inside the nitrogen glovebox.

We observe a small blue shift of approximately 15 meV in the PL peak when the MACl concentration is increased from 0 to 0.3 mol/dm^3 (see figure below and Fig. S2). Beyond this concentration, both the PL peak position and the bandgap remain essentially unchanged, and the FWHM of the PL spectra is constant within experimental error. These observations indicate that above 0.3 mol/dm^3, neither MA^+ nor Cl^- is significantly incorporated into the perovskite lattice and thus does not induce structural modifications.

This invariance in PL response beyond 0.3 mol/dm^3 strongly supports our interpretation that the films are compositionally similar across the series, and that the main variable influencing their degradation behavior is the change in crystallographic orientation rather than a change in material composition.

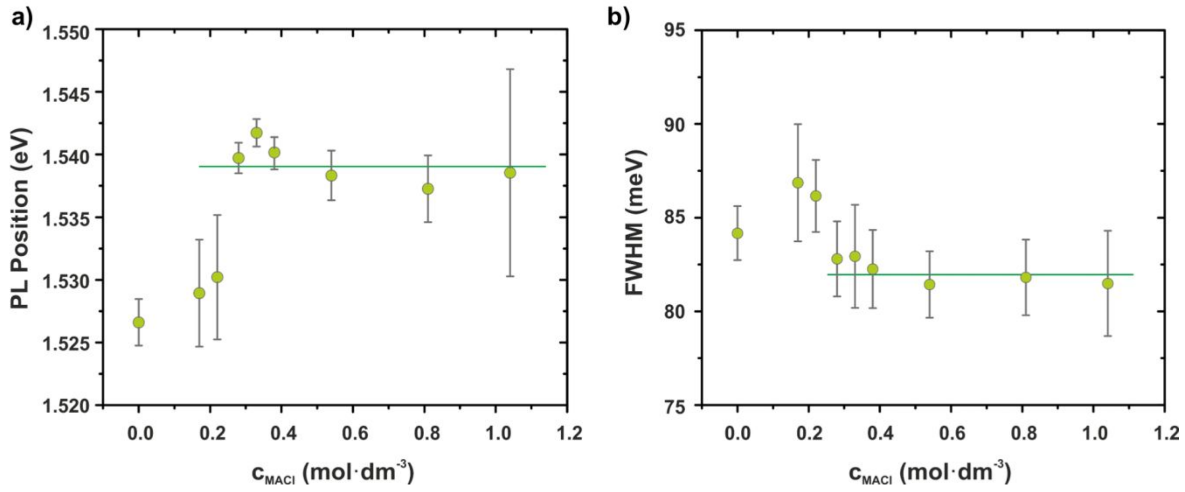

Fig. S2: PL peak position (a) and full width at half maximum (b) as a function of the molar concentration of MACl in MAFA *thin films. The green line is the guide for the eyes.*

We also added the following paragraph into the main text of the manuscript:

Additionally, PL mapping over 40 × 40 μm^2 areas reveals a small blue shift of approximately 15 meV in the PL peak when the MACl concentration is increased from 0 to 0.3 mol/dm^3. At higher concentrations, however, both the PL peak position and the bandgap remain unchanged, and the FWHM of the PL spectra is constant within experimental error. This invariance in PL response indicates that neither MA^+ nor Cl^- are significantly incorporated into the perovskite lattice beyond 0.3 mol/dm^3. Consequently, the perovskite films across the series are compositionally similar, and the observed differences in stability can be

attributed primarily to changes in crystallographic orientation rather than to variations in material composition.

3) **Clarification is also needed regarding the conditions and protocols for photoluminescence measurements. In particular, as the probed materials are highly sensitive to air and can degrade quite quickly, it is important to carry out measurements as soon as possible after deposition, and before any degradation occurs. Can you give some precisions? (and also precise if the samples do not degrade upon luminescence measurements)**

Thank you for your question. Indeed, perovskites are sensitive to air. During this study, sets of identical samples were deposited and characterized using various techniques, including PL and PLQY. Sample deposition was carried out in a nitrogen glovebox, and both PL and PLQY measurements were performed in a separate nitrogen glovebox. Samples were transferred hermetically (samples packed inside aluminum foil in an airtight jar) between gloveboxes in a nitrogen atmosphere, avoiding exposure to light and ambient atmosphere. The PL spectra were acquired using a Renishaw InVia confocal spectrometer operating inside the nitrogen glovebox. During the PL measurements, the samples showed no evidence of degradation under excitation at 532 nm.

We also added the following paragraph to the Characterization section of the manuscript:

The photoluminescence (PL) spectra were acquired using a Renishaw InVia confocal spectrometer equipped with 532 nm laser excitation and a Leica 50× objective inside a nitrogen glovebox, providing PL mapping over 40 × 40 μm^2 areas. To ensure the samples remained pristine, they were hermetically transferred between gloveboxes, sealed in aluminum foil within an airtight jar under a nitrogen atmosphere, thereby avoiding exposure to light and ambient air. During the PL measurements, the samples showed no signs of degradation under 532 nm excitation.

4) **The description of X-ray diffraction patterns could be somewhat developed. Indeed, you focus on the predominance of {100} related peaks and disappearance of other peaks when increasing CMACl beyond 0.38. However, one can also observe that at the same time the peaks related to the {100} family become strongly asymmetric with a pronounced shoulder on the high 2q side of the peak. I think that it is important to comment on that and propose some explanation because this might also be an indicator of the increased sensitivity to degradation of the samples under ambient conditions, not only just the predominance of {100} facets.**

The shoulders of the {100} and {200} peaks in Fig. 2 are exaggerated due to the logarithmic intensity scale. On a linear scale, the peaks display a standard shape that can be well described by pseudo-Voigt or Lorentzian profiles, and they are essentially symmetric. The apparent asymmetry of the shoulders is an instrumental artefact, created by the use of a β-filter in the diffracted beam, which decreases intensity at the low-angle side of the peak and produces a step in the shoulder. The logarithmic scale was chosen to highlight weaker peaks that are hardly visible on a linear scale; however, it also distorts the peak shape and accentuates such instrumental artefacts.

We also added the following sentences to the main manuscript:

The shoulders of the {100} and {200} peaks in Fig. 2 are exaggerated due to the logarithmic intensity scale. On a linear scale, the peaks display a standard shape that can be well described by pseudo-Voigt or Lorentzian profiles, and they are essentially symmetric.

5) **Also, there are peaks at low 2q values (<15 degrees) marked with stars. What is the origin of these peaks? What is the meaning of the marked stars? You should add information on that (at least in the figure caption)**

We thank the reviewer for pointing this out. The peaks marked with stars correspond to the hexagonal δ-$FAPbI_3$ phase, as indicated in the legend of Fig. 2a. To avoid any ambiguity, we have now explicitly mentioned this sentence in the figure caption as follows:

Key reflections corresponding to α-$FAPbI_3$ and δ-$FAPbI_3$ impurity phases are indexed.

6) **Do you have any information on possible strains in the material and their relation to stability Issues?**

We thank the reviewer for this question. We attempted to evaluate the strain in the series of samples (values are summarized in the table below). However, the calculated strain values are largely comparable to the associated experimental uncertainties, and no consistent trend could be identified across the samples. On this basis, we conclude that strain is not significant (or at least not clearly detectable by XRD in our case) and therefore does not show a clear correlation with stability.

**Sample Number**	C_{MACl}	**Lattice Strain (da/a * 1e3)**
1	0	0.35(61)

2	0.17	0.12(20)
3	0.22	0.50(39)
4	0.28	0.60(55)
5	0.33	0.59(62)
6	0.38	1.63(64)
7	0.54	0.05(13)
8	0.81	1.08(51)
9	1.04	0.70(22)

7) **Stability is only considered from visual pictures of the samples after 72 hours. There is no indication about structural and photoelectrical changes of the material. Is the evolution linked to the appearance of the d phase or to PbI2? The d phase should be visible in the X-ray diffraction patterns. Did you investigate the samples through XRD and PL after the 72 hours storage period?**

We thank the reviewer for this comment. To address this point, we analyzed a degraded (yellow) sample after 72 h of ambient storage using XRD. The analysis shows that the perovskite film transforms primarily into the hexagonal δ-$FAPbI_3$ phase. Phase quantification indicates that the degraded film consists of approximately 85 volume% δ-$FAPbI_3$ and 15 volume% residual α-$FAPbI_3$. These results confirm that the instability observed under ambient conditions is mainly associated with the α → δ phase transition.

We have added the spectra of the sample after 72 h of air exposure (yellow sample) for the sample with c_{MACl} 0.81 in Fig. 2a.

We also added the following sentences to the main manuscript:

In contrast, the XRD pattern of the sample (c_{MACl} = 0.81) degraded in air after 72 h of exposure shows a transition from the pure α-phase to approximately 85 volume% δ-phase and 15 volume% residual α-phase.

8) **One of the main concern is the distinction between performance and stability. Indeed, PL measurements are an indicator of quasi-Fermi level splitting, which can be**

linked to the open-circuit voltage in cells. This is related to the potential performance of solar cells. So the direct comparison of PL and XRD establishes a link between the structural properties, in particular the presence of (100) facets, and performance. On the other hand, you observe that samples prepared with larger MaCl content in the solution exhibit stronger visual degradation. But the conclusion drawn here is not straightforward and mixes performance and stability. Larger grains are normally supposed to be favourable to performance due to less grain boundaries and therefore less recombination activity. The presented results do not agree with this general thought, since the grain size continuously increases with the MaCl content. It is maybe possible to discuss the measurement results and this usual guess in terms of surface states. One could also relate the higher sensitivity of (100) surfaces to surface activity. In other words, could your combined measurements point toward both a larger surface recombination detrimental to performance (due to higher surface defect density or to higher capture cross sections of the surface defects) and a larger surface "activity" or sensitivity to external factors detrimental to stability.

We thank the reviewer for highlighting the distinction between optoelectronic performance and environmental stability. Indeed, PL measurements primarily probe quasi-Fermi level splitting, which correlates with potential solar cell performance, whereas visual degradation under ambient conditions reflects long-term stability.

In our study, while larger grains reduce grain boundary density and initially enhance PL (improving performance), we observe that films with high MACl content — which favor larger grains with dominant {100} crystallographic orientation — degrade more rapidly. We attribute this to facet-dependent surface effects: {100} facets exhibit higher surface defect densities and enhanced surface reactivity, which can simultaneously lead to (i) increased non-radiative recombination under illumination (reducing PL in the long term) and (ii) greater susceptibility to environmental factors such as moisture and oxygen.

Thus, our combined PL and XRD measurements point toward a dual effect of {100} surfaces: they are beneficial for reducing grain boundary recombination in moderate concentrations but also more "active" or sensitive at the surface, which is detrimental to long-term stability.

We have revised the Results and Discussion section to clarify this distinction and include a discussion of surface activity effects.

Was: Taken together, the decrease in PLQY, which reflects increased defect density, and the ambient-air stability experiments result indicate that {100} crystallographic orientation

compromises the stability of MAFA perovskite films. It appears that films with a dominant {100} orientation may degrade more rapidly due to a combination of more reactive surface chemistry[33], increased defect density[34], higher moisture affinity[35], and enhanced ion migration[36], all of which contribute to instability. However, a definitive explanation requires further detailed investigation, including microscopic analysis and additional advanced characterization techniques.

Now: While larger grains typically reduce grain boundary density and thereby enhance optoelectronic performance, as evidenced by higher PLQY values, our results indicate that films with dominant {100} facets exhibit accelerated environmental degradation. This facetdependent behavior arises from two complementary effects: (i) {100} surfaces possess higher surface defect densities, which can increase non-radiative recombination under illumination, and (ii) these surfaces are more chemically "active," rendering them more susceptible to external factors such as moisture and oxygen.33–36 Consequently, PLQY measurements primarily reflect the intrinsic potential for high photovoltaic performance, whereas ambient degradation highlights the facet-dependent susceptibility of the material. This distinction underscores the importance of considering both grain size and crystallographic orientation when optimizing MAFA perovskite thin films for stable, high-efficiency devices.

9) In the conclusion, the range of cMACl allowing to achieve PLQY larger than 8% is stated as from 0.54 and 0.81 mol dm-3. This is not in agreement with the results of Fig. 3 and with the abstract and should be corrected to "from 0.28 to 0.38 mol dm-3".

Thank you to the reviewer for noticing this. Indeed, it should be corrected to "from 0.28 to 0.38 mol·dm^{-3}." This correction has been made in the main manuscript.

Reviewer #2

Original review: *In this study from Khytko and coworkers the impact of grain size and crystallographic orientation on the stability and optoelectronic properties of FAMA perovskite thin films was investigated.*

By varying the amount of MACl in the spin-coating solvent the grain sizes could be gradually increased. Color changes demonstrate that larger grains with a dominant {100} orientation degrade more rapidly under ambient conditions than isotropic aligned samples with small grains.

At present studying the stability and revealing the degradation mechanism are very important topics for the use of perovskite materials. As such the present manuscript is of interest for JPCL. At the same time the main message of the paper is for me not very well argued. For this reason the manuscript is at this stage not appropriate for JPCL. The hesitations I have are as follows:

Dear Reviewer 2, thank you for your time and the valuable comments, questions, and suggestions. The answers are listed below.

1) **All effects are related to the preferential orientation of the PVK, however in current literature the presence of MA negatively affects the stability. The composition of the PVKs with different amounts of MACl also changes which could also explain the degradation. Also Cl- could negatively affect the stability.**

We thank the reviewer for highlighting the potential influence of MA^+ and Cl^- on film stability. We performed PL mapping on all samples over 40 × 40 μm^2 areas using a Renishaw InVia confocal spectrometer operating inside the nitrogen glovebox in order to address this question.

We observe a small blue shift of approximately 15 meV in the PL peak when the MACl concentration is increased from 0 to 0.3 mol/dm^3 (see figure below and Fig. S2). Beyond this concentration, both the PL peak positions remain essentially unchanged, and the FWHM of the PL spectra is constant within experimental error. These observations indicate that above 0.3 mol/dm^3, neither MA^+ nor Cl^- is significantly incorporated into the perovskite lattice and thus does not induce structural modifications.

This invariance in PL response strongly supports our interpretation that the films are compositionally similar above 0.3 mol/dm^3, and that the main variable influencing their degradation behavior is the change in crystallographic orientation rather than a change in material composition.

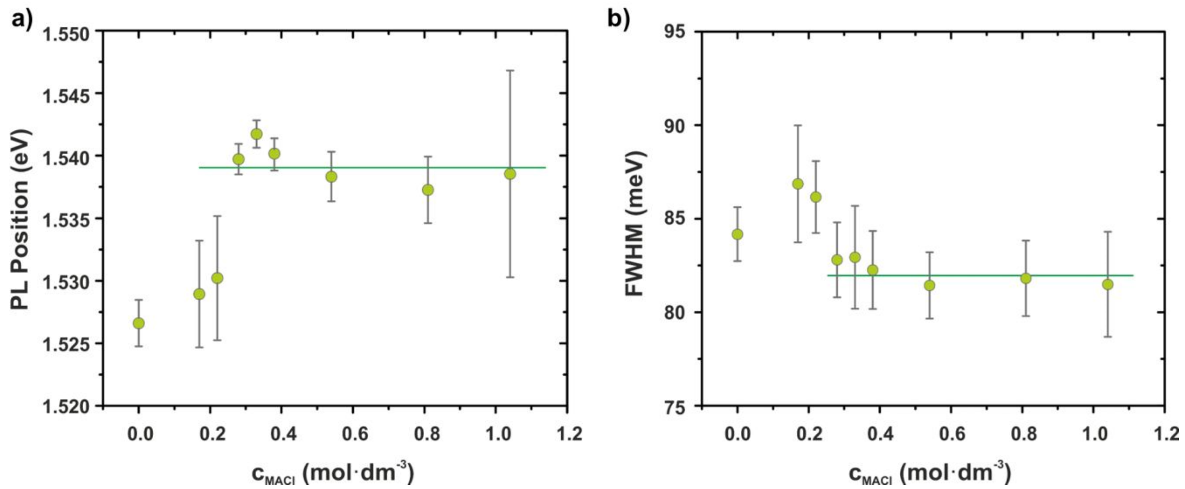

Fig. S2: PL peak position (a) and full width at half maximum (b) as a function of the molar concentration of MACl in MAFA *thin films. The green line is the guide for the eyes.*

Indeed, previous studies have shown that high MA^+ content can reduce the long-term stability of $FAPbI_3$-based perovskites due to its volatility and tendency to form defects at grain boundaries. (ref.1) Similarly, residual Cl^- has been reported to affect crystallization kinetics and potentially accelerate degradation under ambient conditions. (ref.2)

In our study, however, the incorporated MA^+ content in the final perovskite layers is expected to be very low (<10% of the initial precursor amount), consistent with the observations of Zheng et al. (ref.3) and the known volatility of MA^+ during thermal annealing. Likewise, the residual Cl^- fraction is minimal, as most Cl^- is evaporated during the annealing process, consistent with previous reports (ref.2). Therefore, while MA^+ and Cl^- may have minor contributions, our combined PLQY, XRD, and stability measurements indicate that the dominant factor controlling degradation is the preferential {100} orientation, which correlates with both higher defect density and increased surface sensitivity to environmental factors.

Most of the Cl^- in MACl is volatilized during the initial high-temperature annealing step, while any residual surface-bound chloride is further removed during a subsequent $100\ °C$ post-heat treatment. As a result, after 15 minutes at $150\ °C$ followed by 30 minutes at $100\ °C$, the remaining chloride is expected to be negligible, present only in trace amounts. This is consistent with literature reports showing that MACl-treated $FAPbI_3$ films lose the bulk of their chloride within the first few minutes of thermal annealing, although small amounts can persist temporarily at grain surfaces, grain boundaries, or interstitial sites. The low-temperature postheat treatment specifically targets these residual chlorides,

promoting the decomposition and volatilization of any remaining MACl, thereby ensuring that the final perovskite layer contains minimal chloride and that its contribution to the film composition is insignificant.

In short, the data suggest that crystallographic orientation plays the primary role in the observed degradation behavior.

Ref. 1 - Wang, C.; He, B.; Fu, M.; Su, Z.; Zhang, L.; Zhang, J.; Mei, B.; Gao, X. Influence of MACl on the Crystallization Kinetics of Perovskite via a Two-Step Method. *Crystals* **2024**, *14* (5), 399. https://doi.org/10.3390/cryst14050399.

Ref. 2 - Kang, D.-H.; Lee, S.-U.; Park, N.-G. Effect of Residual Chloride in $FAPbI_3$ Film on Photovoltaic Performance and Stability of Perovskite Solar Cell. *ACS Energy Lett.* **2023**, *8* (5), 2122–2129. https://doi.org/10.1021/acsenergylett.3c00568.

Ref. 3 - Zheng, D.; Chen, F.; Rager, M.; Gollino, L.; Zhang, B.; Pauporté, T. What Are Methylammonium and Solvent Fates upon Halide Perovskite Thin-Film Preparation and Thermal Aging? *Adv. Mater. Interfaces* **2022**, *9* (32). https://doi.org/10.1002/admi.202201436.

We also added the following paragraph into the main text of the manuscript:

Additionally, PL mapping over 40 × 40 μm^2 areas reveals a small blue shift of approximately 15 meV in the PL peak when the MACl concentration is increased from 0 to 0.3 mol/dm^3. At higher concentrations, however, both the PL peak position and the bandgap remain unchanged, and the FWHM of the PL spectra is constant within experimental error. This invariance in PL response indicates that neither MA^+ nor Cl^- are significantly incorporated into the perovskite lattice beyond 0.3 mol/dm^3. Consequently, the perovskite films across the series are compositionally similar, and the observed differences in stability can be attributed primarily to changes in crystallographic orientation rather than to variations in material composition.

2) **With no or little amount of MACl the gamma phase is observed in the XRD patterns. It could also be argued that a small amount of gamma phase suppresses degradation.**

We thank the reviewer for this comment. In our XRD patterns, the peak observed around 12° corresponds to the hexagonal δ-$FAPbI_3$ phase, and no γ-phase was detected. Our observations are consistent with the literature (ref.1). The γ-phase of $FAPbI_3$ is a hexagonal polymorph that typically forms under specific conditions such as low temperature or high

pressure. While the γ-phase is thermodynamically more stable and could, in principle, suppress degradation, its diffraction peaks (~2θ = 12.7°, 25.5°, 38.7°) often overlap with PbI_2 peaks, complicating unambiguous identification in our films. We are somewhat uncertain whether the reviewer is specifically inquiring about the presence of the δ-$FAPbI_3$ phase, which is significant in our XRD results for the degraded sample (Fig. 2a), confirming that the instability observed under ambient conditions is mainly associated with the α → δ phase transition.

Ref. 1 - Cuzzupè, D. T.; Ünlü, F.; Lê, K.; Bernhardt, R.; Wilhelm, M.; Grosch, M.; Weißing, R.; Fischer, T.; Van Loosdrecht, P. H. M.; Mathur, S. Thermally-Induced Drift of A-Site Cations at Solid–Solid Interface in Physically Paired Lead Halide Perovskites. *Sci. Rep.* **2022**, *12* (1), 10241. https://doi.org/10.1038/s41598-022-14452-y.

3) **For PVK's with a large amount of MACl the XRD peaks for the (100) and (200) show a very broad base. What causes this? Could that be the factor of importance for the stability?**

We thank the reviewer for this observation. The apparent broad base of the {100} and {200} peaks in Fig. 2a is an artefact resulting from the logarithmic intensity scale used for plotting. On a linear scale, the peaks display a standard symmetric shape that can be well described by pseudo-Voigt or Lorentzian functions. The logarithmic representation was chosen in order to highlight weaker diffraction peaks that would otherwise be barely visible, but it also exaggerates the low-intensity base and distorts the apparent peak shape. Therefore, the broad base is not an intrinsic feature of the material and is not related to stability.

4) **What are the resulting compositions for the different additions in the PVK film? (ratio MA to FA? Cl?)**

We thank the reviewer for raising this important point regarding the resulting compositions of the perovskite films. Indeed, determining the precise stoichiometry after film formation remains a well-known challenge in this field. We attempted to characterize the composition as well as ratio MA to FA using NMR; however, the results could not be reliably interpreted in this case.

Despite the large excess of MACl in the precursor solution, the amount of MA^+ incorporated into the final perovskite lattice is strongly reduced during crystallization and particularly during thermal annealing, due to the volatile nature of MA^+. Zheng et al. (ref.1) demonstrated that even when using precursor solutions with significantly higher MACl

contents, the residual MA^+ fraction stabilizes at only ~2–3 mol% of the A-site cations after optimized annealing. Consistent with this, we assume that in our films the incorporated MA^+ content remains below 10% of the initial precursor amount. This strongly supports our conclusion that the final layers contain only a very minor fraction of MA^+, and that the observed effects are dominated by structural orientation rather than by a major change in bulk composition.

Most of the Cl^- in MACl is volatilized during the initial high-temperature annealing step, while any residual surface-bound chloride is further removed during a subsequent 100 °C post-heat treatment. As a result, after 15 minutes at 150 °C followed by 30 minutes at 100 °C, the remaining chloride is expected to be negligible, present only in trace amounts. This is consistent with literature reports showing that MACl-treated $FAPbI_3$ films lose the bulk of their chloride within the first few minutes of thermal annealing, although small amounts can persist temporarily at grain surfaces, grain boundaries, or interstitial sites (ref.2).

For clarity, we report both the initial precursor solution composition and the use of MACl additives (Table S1), as well as the detailed sample preparation procedure described in Section 2.1 of the main manuscript.

Furthermore, as evident from the answer to Question 1 and the graph below, both the PL peak position and the FWHM exhibit saturation behavior. These observations indicate that above 0.3 mol/dm^3, neither MA^+ nor Cl^- is significantly incorporated into the perovskite lattice and thus does not induce structural modifications.

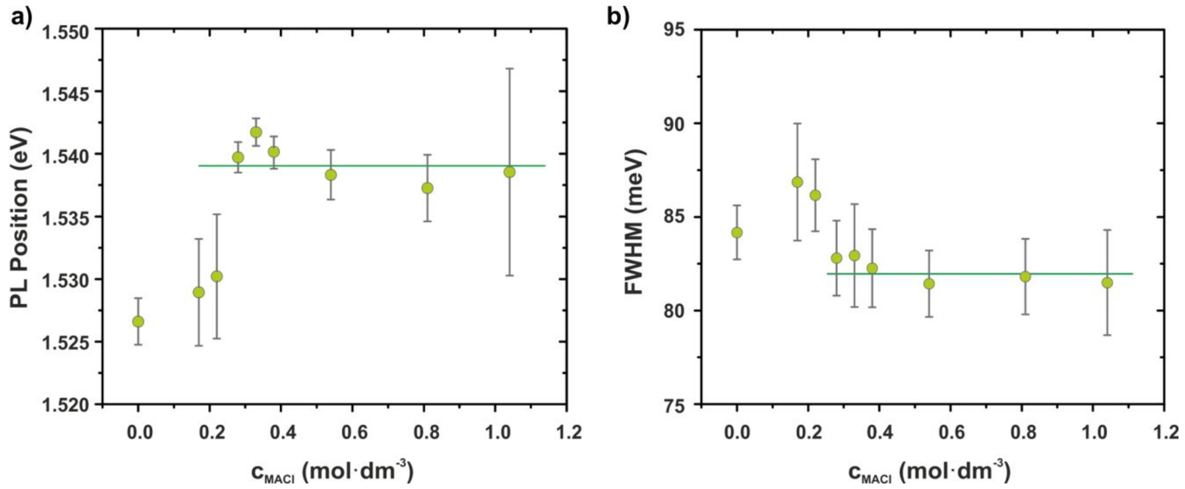

Ref. 1 - Zheng, D.; Chen, F.; Rager, M.; Gollino, L.; Zhang, B.; Pauporté, T. What Are Methylammonium and Solvent Fates upon Halide Perovskite Thin-Film Preparation and Thermal Aging? *Adv. Mater. Interfaces* **2022**, *9* (32).https://doi.org/10.1002/admi.202201436.

Ref. 2 - Kang, D.-H.; Lee, S.-U.; Park, N.-G. Effect of Residual Chloride in $FAPbI_3$ Film on Photovoltaic Performance and Stability of Perovskite Solar Cell. *ACS Energy Lett.* **2023**, *8* (5), 2122–2129. https://doi.org/10.1021/acsenergylett.3c00568.

5) **The degradation assessment is very basic. Just having a couple of absorption spectra measured at different days could already provide a lot of detailed information.**

We thank the reviewer for this suggestion. While UV–Vis absorption spectroscopy is indeed a valuable tool for assessing degradation, our study was not designed to track this systematically over time. The primary focus of our work is to investigate how freshly crystallized perovskite grains degrade under ambient air and how crystallographic orientation influences this process. We refer to question 6 for the more detailed degradation assessment.

6) **Recording the XRD during degradation could also confirm the claims of the manuscript**

We thank the reviewer for this comment. To address this point, we analyzed a degraded (yellow) sample after 72 h of ambient storage using XRD. The analysis shows that the perovskite film transforms primarily into the hexagonal δ-$FAPbI_3$ phase. Phase quantification indicates that the degraded film consists of approximately 85 volume% δ-$FAPbI_3$ and 15 volume% residual α-$FAPbI_3$. These results confirm that the instability observed under ambient conditions is mainly associated with the $\alpha \rightarrow \delta$ phase transition.

We have added the spectra of the sample after 72 h of air exposure (yellow sample) for the sample with c_{MACl} 0.81 in Fig. 2a.

We also added the following sentences to the main manuscript:

In contrast, the XRD pattern of the sample (c_{MACl} = 0.81) degraded in air after 72 h of exposure shows a transition from the pure α-phase to approximately 85 volume% δ-phase and 15 volume% residual α-phase.

7) **Muscarella et al have a relevant paper on this topic. (J. Phys. Chem. Lett. 2019,**

10, 6010–6018)

We thank the reviewer for bringing the relevant work by Muscarella et al. (J. Phys. Chem. Lett. 2019, 10, 6010–6018) to our attention. In that study, the authors investigated the influence of crystal orientation and grain size on the optoelectronic properties of $MAPbI_3$ perovskite films and concluded that orientation alone was not the dominant factor in determining performance. While our results similarly highlight that orientation cannot be the only descriptor of performance, we also find that the preferential formation of {100} facets correlates strongly with reduced environmental stability. By combining PL and stability measurements, our study emphasizes that facet-dependent effects may play a dual role: having only a limited impact on optoelectronic performance under short-term measurements, but exerting a strong influence on long-term material stability. We have now cited and briefly discussed Muscarella et al. in the revised manuscript.

jz-2025-027282.R2

Name: Peer Review Information for "Grain Size-Dependent Stability and Crystallographic Orientation Effects in MAFA Perovskite Thin Films"

Second Round of Reviewer Comments

Reviewer: 1

Comments to the Author

Thank you for taking my comments and suggestions into consideration and providing reasonable and satisfactory answers to my questions. The revised version of the manuscript includes clarifications and new arguments to address the points raised in my initial report.

In conclusion, I consider that the manuscript is now suitable for publication in the Journal of Physical Chemistry Letters.

Reviewer: 2

Comments to the Author

In the revised version of the paper of Khytko et al some of the remarks of both reviewers were included. Unfortunately most of the comments haven't been addressed in a convincing way by e.g. an additional measurement, which in the end might compromise the impact of the paper. It is up the editor to take a decision on this issue.

Author's Response to Peer Review Comments:

Dear Editor,

We would like to thank you and the reviewers for your careful evaluation of our revised manuscript and for the positive and encouraging feedback. We are grateful for the opportunity to make the final non-scientific corrections required for acceptance.

In response to the editorial request:

1. The TOC graphic has been resized to fit within the required dimensions (3.25 × 1.75 inches; 8.25 × 4.45 cm), and all text has been checked for clarity and legibility at this scale.
2. The reference 35 has been updated to include complete bibliographic information, including author names, article title, journal name, publication year, and first page number (If we understood the request correctly).

We confirm that no scientific changes have been made to the manuscript.

We truly appreciate the reviewers constructive feedback, which significantly improved the quality and clarity of our work, and we thank you for considering our manuscript for publication in The Journal of Physical Chemistry Letters.

Sincerely,

Mykhailo Khytko

Research and Development Specialist

Department of Thin Films and Nanostructures

Institute of Physics of Czech Academy of Sciences

Prague, Czechia

+420606158738

khytko@fzu.cz
